# The significance of long chain non-coding RNA signature genes in the diagnosis and management of sepsis patients, and the development of a prediction model

**DOI:** 10.3389/fimmu.2024.1450014

**Published:** 2024-12-12

**Authors:** Yong Bai, Jing Gao, Yuwen Yan, Xu Zhao

**Affiliations:** ^1^ Intensive Care Unit, Hubei University of Medicine, Renmin Hospital, Shiyan, Hubei, China; ^2^ Department of Gastroenterology 3, Hubei University of Medicine, Renmin Hospital, Shiyan, Hubei, China; ^3^ Institute of Clinical Medicine, Hubei University of Medicine, Renmin Hospital, Shiyan, Hubei, China

**Keywords:** the significance of long chain non-coding RNA, signature genes, sepsis, diagnosis and management, prediction model

## Abstract

**Background:**

Sepsis is a life-threatening organ dysfunction condition produced by dysregulation of the host response to infection. It is now characterized by a high clinical morbidity and mortality rate, endangering patients’ lives and health. The purpose of this study was to determine the value of Long chain non-coding RNA (LncRNA) RP3_508I15.21, RP11_295G20.2, and LDLRAD4_AS1 in the diagnosis of adult sepsis patients and to develop a Nomogram prediction model.

**Methods:**

We screened adult sepsis microarray datasets GSE57065 and GSE95233 from the GEO database and performed differentially expressed genes (DEGs), weighted gene co-expression network analysis (WGCNA), and machine learning methods to find the genes by random forest (Random Forest), least absolute shrinkage and selection operator (LASSO), and support vector machine (SVM), respectively, with GSE95233 as the training set and GSE57065 as the validation set. Differentially expressed genes (DEGs), weighted gene co-expression network analysis (WGCNA), boxplot statistical analysis, and ROC analysis by Random Forest, Least Absolute Shrinkage and Selection Operator (LASSO), and Support Vector Machine (SVM) machine learning methods were used to identify characteristic genes and build the Nomogram Prediction model.

**Results:**

GSE95233 yielded a total of 1069 genes, 102 of which were sepsis-related and 22 of which were non-sepsis controls. GSE57065 yielded a total of 899 genes, with 467 up-regulated and 432 down-regulated, including 82 sepsis-related genes and 25 non-sepsis control genes. WGCNA analysis excluded outlier samples, leaving 2,029 genes for relationship analysis between sepsis- and non-sepsis patient-associated LncRNA network representation modules, as well as Wein plots of differential genes versus genes in key modules of weighted co-expression network analysis to analyze gene intersections. Machine Learning found the sepsis-related characteristic LncRNAs RP3-508I15.21, RP11-295G20.2, LDLRAD4-AS1, and CTD-2542L18.1. The datasets GSE95233 and GSE57065 were analyzed using Boxplot against the screened genes listed above, respectively. The p-value between the sepsis and non-sepsis groups was less than 0.05, indicating that anomalies were statistically significant. CTD-2542L18.1 in dataset GSE57065 had an AUC value of 0.638, which was less than 0.7 and did not indicate diagnostic significance, but RP3-508I15.21, RP11-295G20.2, and LDLRAD4-AS1 had AUC values more than 0.7 after ROC analysis. All four sepsis-associated LncRNA ROC analyses in dataset GSE95233 exhibited AUC values more than 0.7, indicating diagnostic significance.

**Conclusion:**

LncRNAs RP3_508I15.21, RP11_295G20.2, and LDLRAD4_AS1 have some utility in the diagnosis and treatment of adult sepsis patients, as well as some reference importance in guiding the diagnosis and treatment of clinical sepsis.

## Introduction

Sepsis is a systemic life-threatening organ dysfunction syndrome with high mortality and severe complications, and despite significant advances in diagnostic and therapeutic approaches to sepsis over the last few decades, the disease continues to be a major life-threatening public health problem globally (1). With the in-depth study of the pathogenesis and therapeutic methods of sepsis, we continue to understand the complexity and diversity of sepsis multi-organ injury, whose pathogenesis has yet to be fully elucidated, and that the immune system plays an important role in the development and progression of sepsis, which primarily includes the activation and regulation of inflammatory cells, the host defense response, and the breakdown of immune tolerance (2–4). Among them, macrophages are one of the most important inflammatory cells. They initiate the inflammatory response and secrete a variety of inflammatory mediators by recognizing and phagocytosing endotoxins released by Gram-negative bacteria. These inflammatory mediators are capable of eliciting systemic inflammatory responses and exerting toxic effects on the visceral organs (5). In addition, immune cells such as T cells and B cells have a role in the development and progression of sepsis by stimulating or restraining the inflammatory response. Furthermore, immunological tolerance breakdown, such as aberrant immune responses to self-antigens or flora, plays a crucial role in the development of sepsis (6).

Recent research has shown that long chain non-coding RNAs (lncRNAs) play a vital role in inflammation and immunological modulation (7, 8). LncRNAs Their function and expression may be associated with the start and progression of sepsis. As a result, the purpose of this research is to investigate the roles and mechanisms of RP3_508I15.21, RP11_295G20.2, and LDLRAD4_AS1 in sepsis and develop a Nomogram prediction model.

## Materials and methods

### Microarray dataset collection and data process the adult sepsis microarray

The adult sepsis microarray dataset was screened from the GEO database with GSE95233 as the training set, which included 102 genes associated with sepsis and 22 genes from the non-sepsis control group. The validation set was GSE57065, which contained 82 sepsis-associated genes and 25 non-sepsis control genes.

### LncRNA-related differentially expressed genes

We transformed the probe into gene symbol in each dataset based on the platform’s annotation file, when there were multiple probes mapped to the same gene symbol; the mean value of probes was selected as the gene expression value. Differentially expressed genes (DEGs) between sepsis and control were analyzed via the “limma package” in R software, with the following cutoff for adjustment: p value < 0.05 and FC (fold changes) >1.5. The intersection of DEGs and LncRNA-related genes was visualized by the Venn plot.

### Weighted gene co-expression network analysis of LncRNAs with associated sepsis

We utilized R’s “clusterProfiler” package to perform weighted gene co-expression network analysis (WGCNA) of LncRNAs linked with sepsis. Through its study, we investigated the biological functions and clinical diagnostic significance of the related LncRNA potential biomarkers in sepsis progression.

### Machine learning algorithms were used to identify LncRNA signature biomarkers related with sepsis, as well as boxplot statistical and ROC analyses

Random Forest, Least Absolute Shrinkage and Selection Operator (LASSO), and Support Vector Machine (SVM) machine learning algorithms were utilized to identify the distinctive genes, as well as boxplot statistics and ROC analysis.

### Building predictive models

Rtudio software was used to do predictive modeling and produce column Nomogram line graphs based on variables in the created clinical model.

### Statistical analysis

GSE95233The diagnostic accuracy of three hub genes was analyzed with receiver operating characteristic curves (ROC) and expressed as the area under the ROC curves (AUROC) and 95% CI. The sensitivity, specificity, positive predictive value (PPV), negative predictive value (NPV),positive likelihood ratio (PLR), and negative likelihood ratio (NLR) for each gene were calculated. The optimal cutoff values of hub genes were obtained when Youden’s index was fixed at the maximum value. Spearman’s rank tests or Pearson correlation coefficient were used to analyze the associations between hub genes and immune cells and immune checkpoint genes.

## Results

### Identification of LncRNA-related degs between sepsis and control

**Figure 1:**
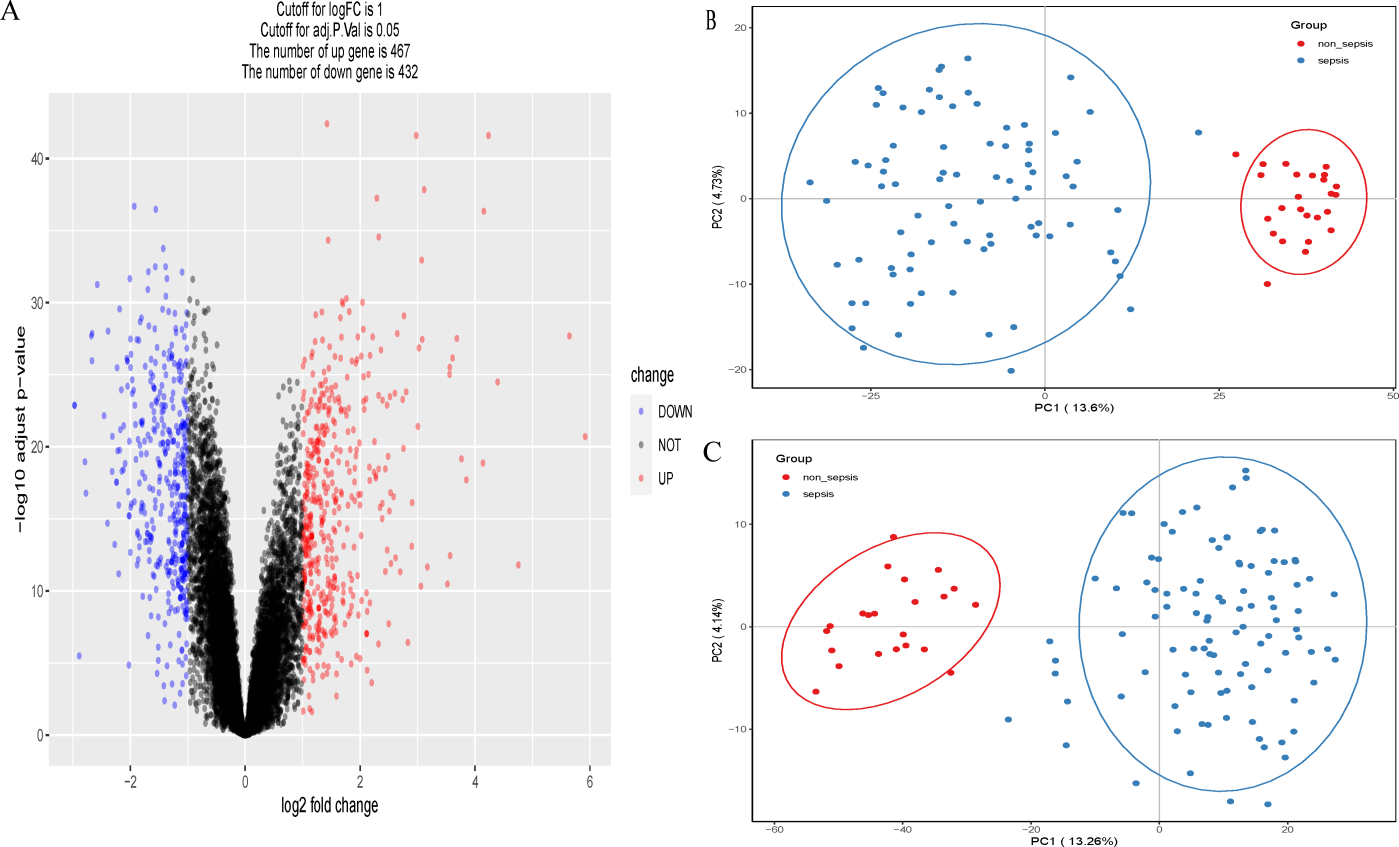
**(A)** Volcano plots of GSE57065. **(B)** Principal component analysis (PCA) scatterplot showing the relationship between septic versus non-septic patients in dataset GSE57065. **(C)** Principal component analysis (PCA) scatterplot showing the relationship between septic versus non-septic patients in dataset GSE95233.

A total of 899 genes were obtained from the adult sepsis microarray dataset GSE57065, 467 of which were up-regulated and 432 of which were down-regulated (**Figure 1A**). Of these, 82 were connected to sepsis and 25 were control genes unrelated to sepsis (**Figure 1B**). The dataset GSE95233 yielded 1069 genes in total, of which 102 were associated to sepsis and 22 were control genes unrelated to sepsis (**Figure 1C**). Genes from both datasets that indicate sepsis and those that do not were subjected to principal component analysis independently.

Weighted gene co-expression network analysis for LncRNAs linked to sepsis. Constructing a weighted gene co-expression network using screened DEG expression data.

### Removal of outlier samples

**Figure 2:**
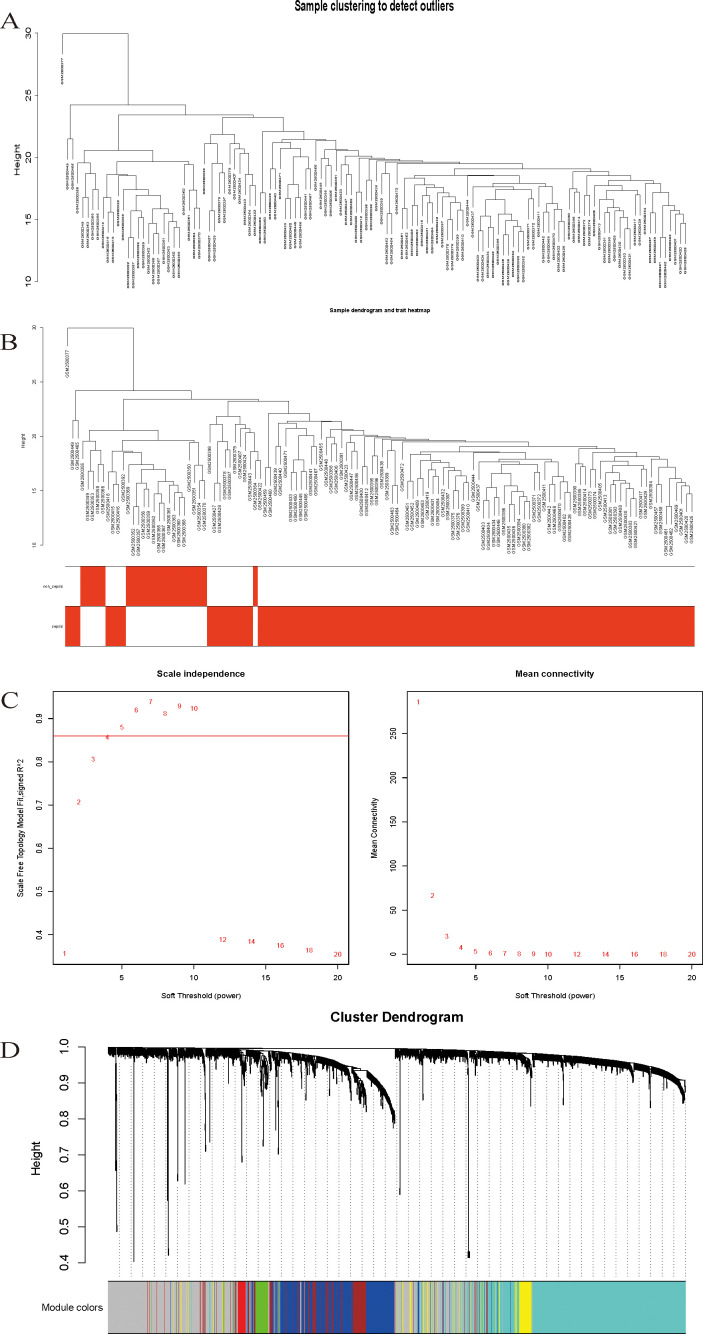
**(A)** Sample clustering to detect outliers. **(B)** Sample dendrogram and trait heatmap. **(C)** Scale-free fit index analysis and average connectivity analysis for different soft threshold powers. **(D)** Hierarchical clustering dendrogram of channel catfish, Ictalurus punctatus, genes with dissimilarity. Each single leaf in the tree represents a single gene, the major tree branches constitute distinct modules and are shown in different colors.

Sample clustering analysis was performed using DEG expression data in the samples to exclude outliers and guarantee the accuracy of the results, and the GSM2500377 samples were finally eliminated (**Figure 2A**), sample dendrogram, and trait heatmap (**Figure 2B**).

### Network construction and modularization

Following the typical selection criterion of 4 for scale-free networks, which is the lowest power of the scale-free topology fitting index of 0.9 (**Figure 2C**), the network was built and the modules were separated to yield 11 valid modules (**Figure 2D**).

**Figure 3:**
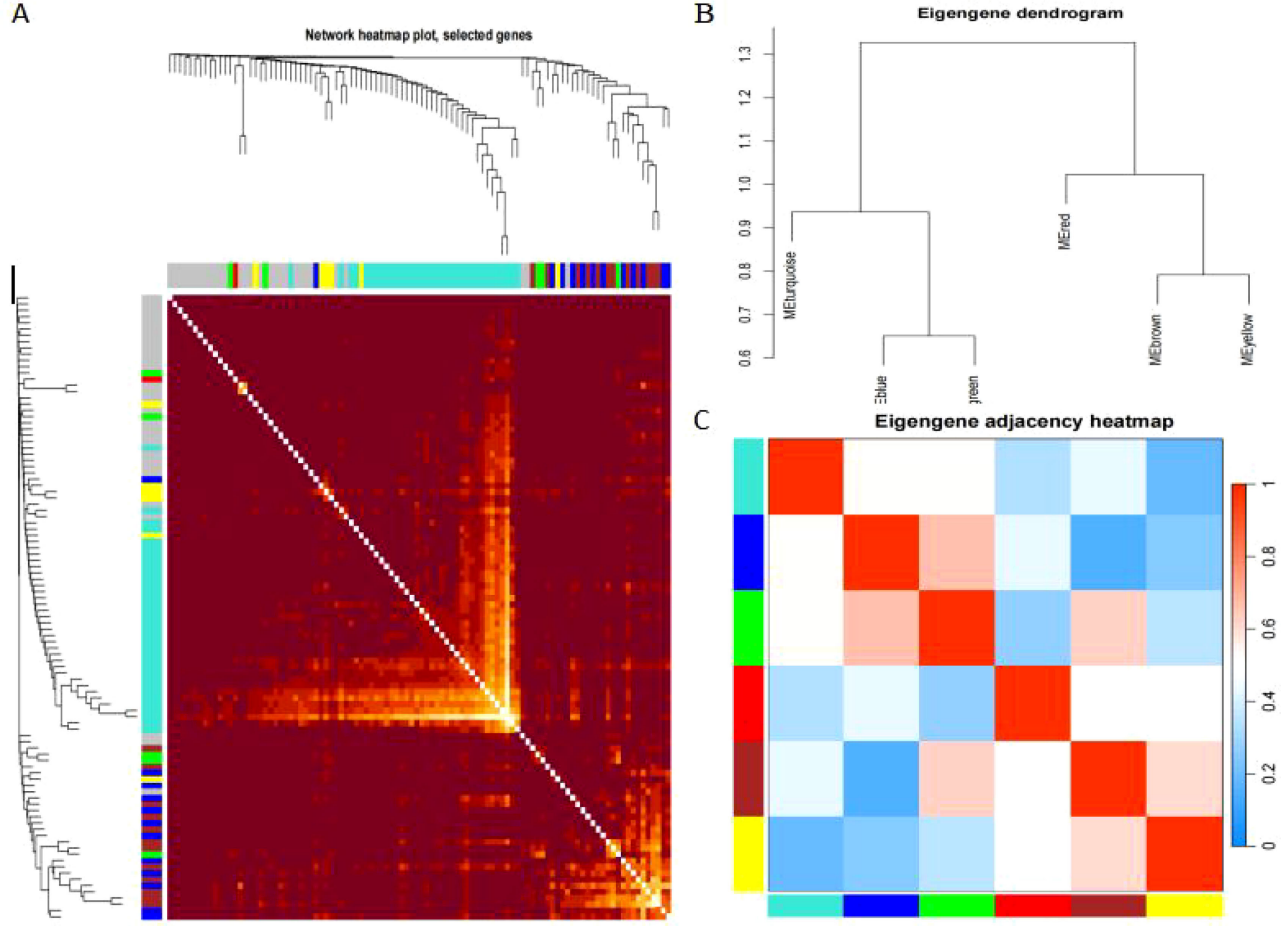
**(A)** Heatmap plot of the gene network in channel catfish, Ictalurus punctatus. The heatmap shows the Topological Overlap Matrix (TOM) among all genes in the analysis. Light color represents high adjacency, and darker color represents low adjacency. The left and top sides indicate the gene dendrogram and module assignment. **(B)** Hierarchical cluster analysis of the genes in different modules. **(C)** connectivity level analysis of the genes in different modules. Within the heatmap, red represents a positive correlation and blue represents a negative correlation. Squares of red color along the diagonal are the meta-modules.

After removing redundant genes, we kept 2,029 genes for additional WGCNA analysis. Our goal was to identify genes that are strongly linked to the development of sepsis and to obtain a thorough understanding of the gene co-expression relationships in sepsis. Gene expression among the six identified modules was relatively independent as illustrated by the topological overlap matrix (TOM) plot of 2029 genes, suggesting that each module was independently validated (**Figure 3A**). The connectivity degree of eigengenes was analyzed to further quantify the similarity of coexpression. The six modules yielded two main clusters, with two sets of three modules each (brown, red and turquoise modules, and black, blue and yellow modules), followed by cluster analysis (**Figure 3B**). The blue and yellow modules, and red and turquoise modules were found to have higher adjacency values based on the heatmap plot of the adjacencies (**Figure 3C**).

**Figure 4:**
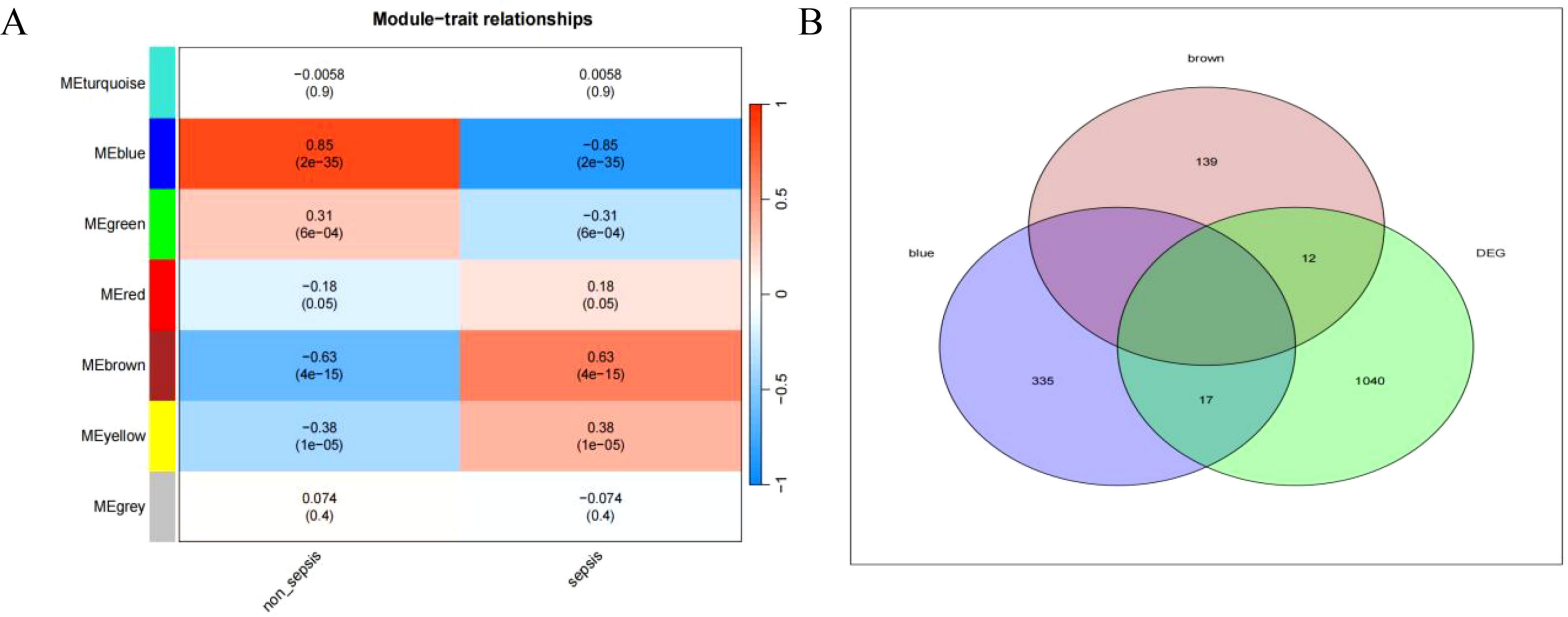
**(A)** depicts the characteristic gene network that represents the relationship between modules; the horizontal coordinate represents the grouping, the vertical coordinate represents the module, and each cell contains the corresponding correlation and P-value; the darker color represents a higher absolute value of P. **(B)** represents the intersection of differential genes with a weighted co-expression network that examines the important module genes.

Relationship between sepsis and non-sepsis patients’ LncRNA network representation modules (**Figure 4A**). Differential gene *vs* weighted co-expression network analysis of essential module genes using a Wayne diagram (**Figure 4B**).

### Machine learning

**Figure 5:**
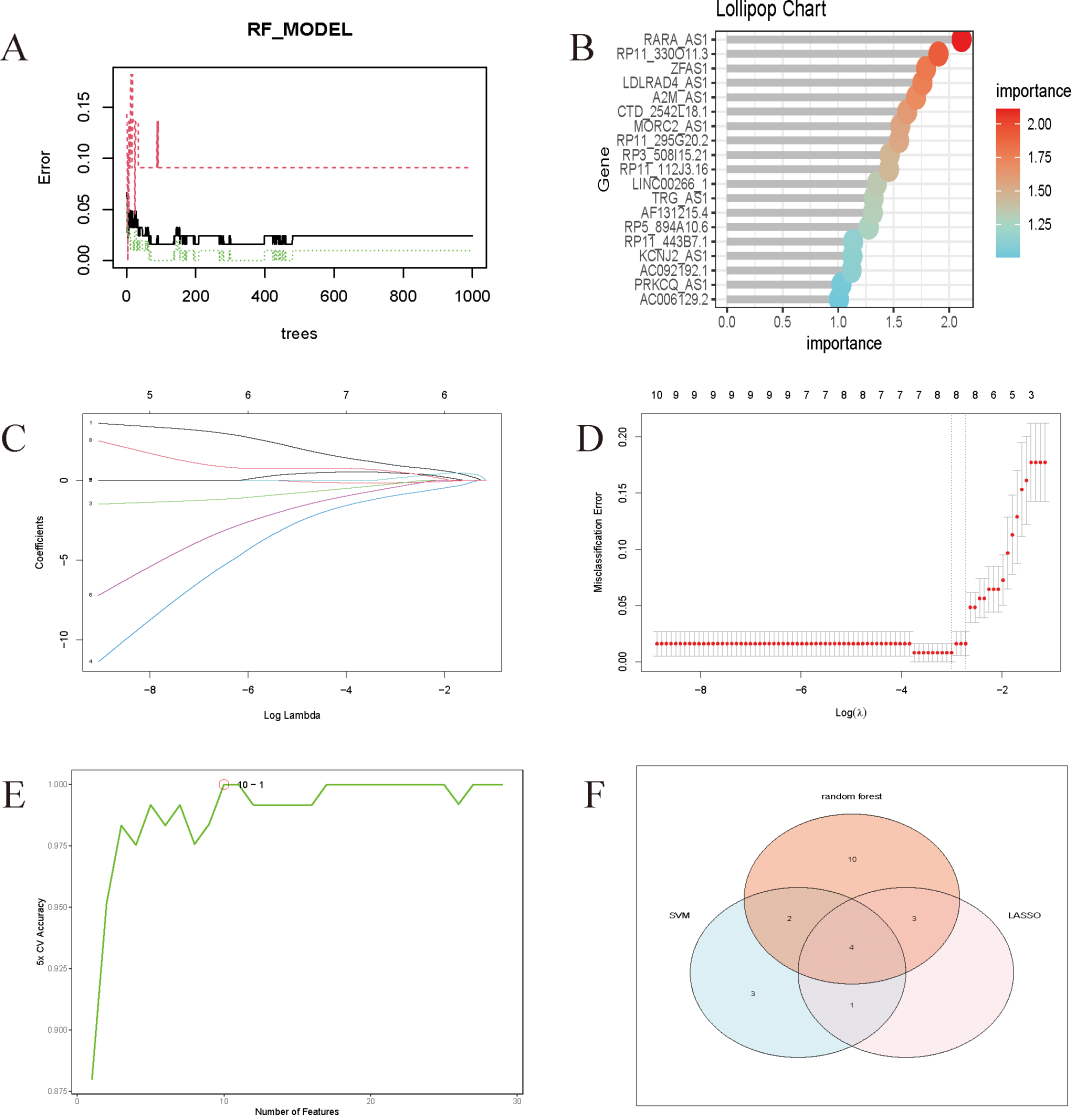
**(A)** Randomized forest trees. The horizontal axis represents the tree and the vertical axis represents the error rate. Red represents sepsis samples, green represents non-sepsis samples, and black represents overall samples. **(B)** Lollipop chart of genes associated with sepsis. The Cross-Validation results of Lasso. **(C)** The coefficients paths at each step of Lasso; **(D)** The Misclassification Error of Lasso. **(E)** The model achieves the highest cross-validation accuracy with around 10 features. Adding more features beyond 10 does not significantly improve the accuracy and might even cause slight fluctuations. **(F)** Wayne diagrams of key genes for 4 Machine Learning methods.

Rtudio software was used to do random forest analysis (**Figures 5A, B**), LASSO analysis (**Figures 5C, D**), and Support Vector Machines analysis (**Figure 5E**) on the screened genes, as well as to generate important genes for various Machine Learning approaches. Wayne plots (**Figure 5F**) were used to detect the LncRNAs linked with sepsis: RP3-508I15.21, RP11-295G20.2, LDLRAD4-AS1, and CTD-2542L18.1.

**Figure 6:**
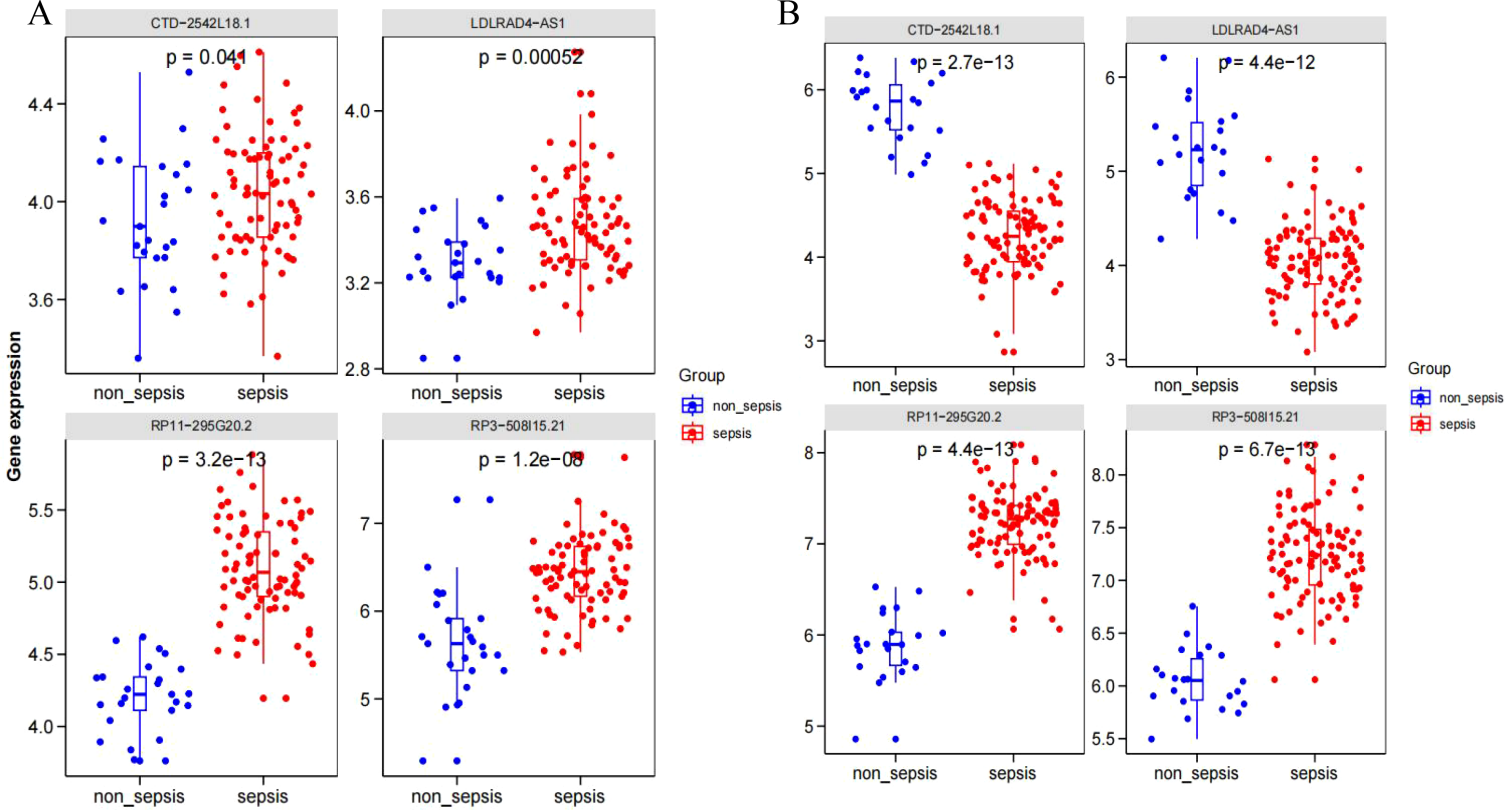
**(A)** Dataset GSE95233; **(B)** Dataset GSE57065.

GSE95233 and GSE57065 datasets were evaluated against the screened sepsis-related characteristic LncRNAs RP3-508I15.21, RP11-295G20.2, LDLRAD4-AS1, and CTD-2542L18.1 using Boxplot analysis, respectively, and p-values of less than 0.05 anomalies existed between sepsis and non-sepsis groups. Statistical significance (**Figures 6A, B**).

**Figure 7:**
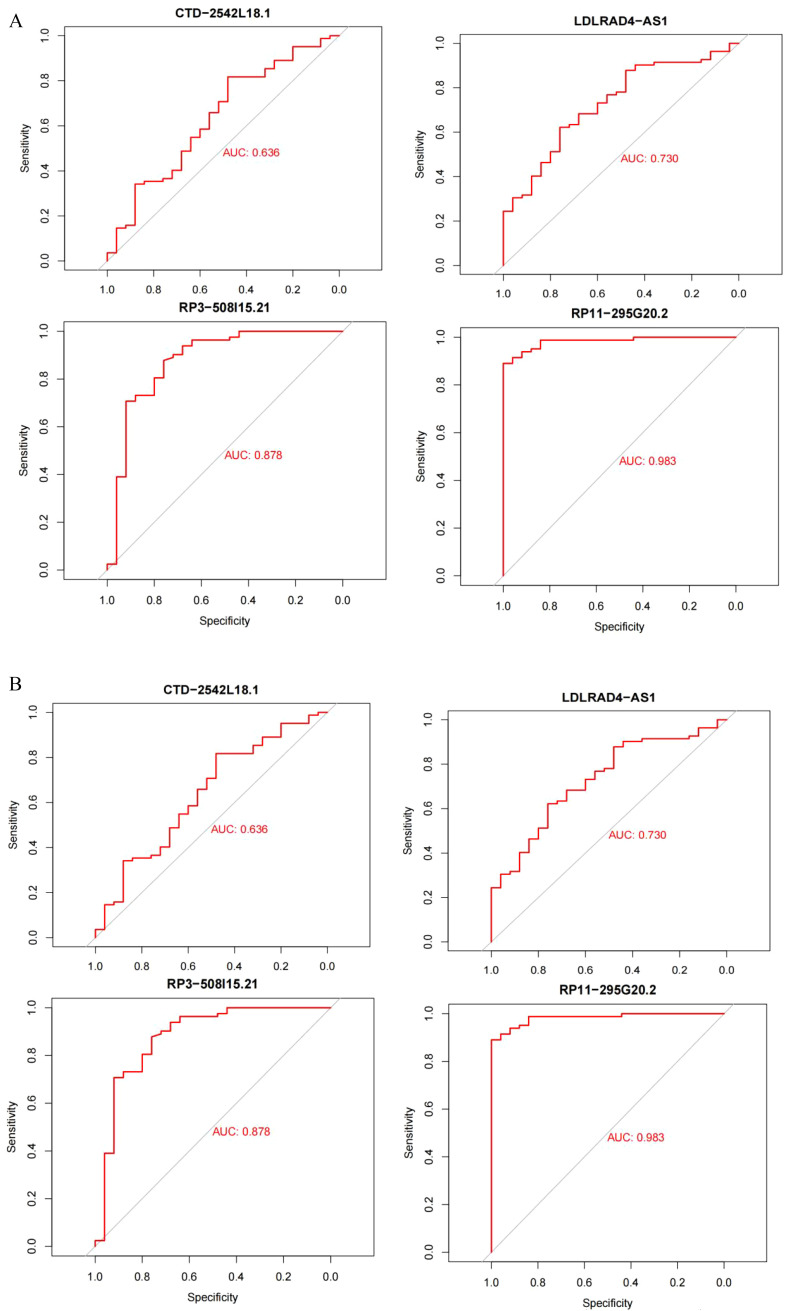
**(A)** Dataset GSE95233; **(B)** Dataset GSE57065.AUC values greater than 0.7 were considered diagnostic.

The datasets GSE95233 and GSE57065 were ROC assessed against the screened sepsis-related characteristic LncRNAs RP3-508I15.21, RP11-295G20.2, LDLRAD4-AS1, and CTD-2542L18.1,with an AUC value greater than 0.7 considered diagnostic. **Figure 7A** depicts the ROC analysis of genes connected to dataset GSE57065, with CTD-2542L18.1 having an AUC value of 0.638, which is less than 0.7 and does not indicate diagnostic significance, and AUC values of RP3-508I15.21, RP11-295G20.2, and LDLRAD4-AS1 being larger than 0.7, indicating diagnostic significance. **Figure 7B** depicts the ROC analysis of the genes linked with the GSE95233 dataset, and the AUC values of the four sepsis-related distinctive LncRNAs were all larger than 0.7,indicating diagnostic relevance.

### Building predictive models

**Figure 8:**
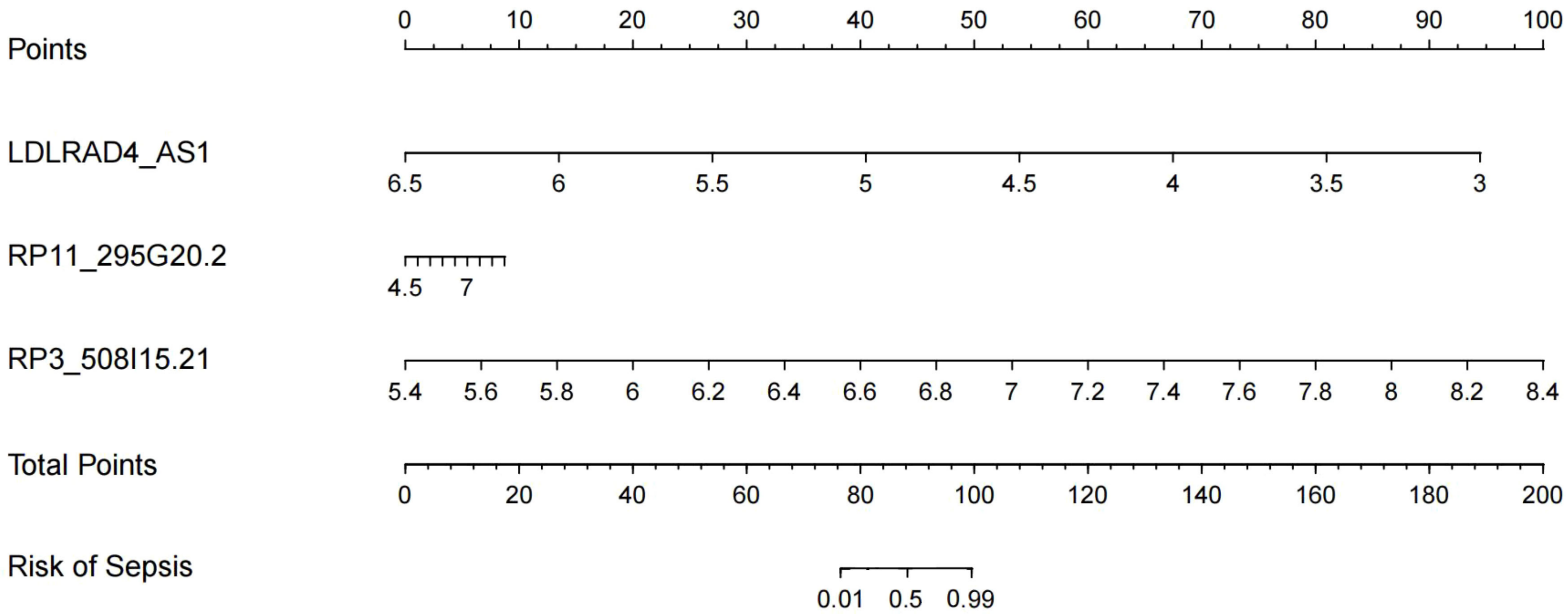
Nomograms model for predicting the risk of sepsis.

After Boxplot and ROC analyses, RP3-508I15.21, RP11-295G20.2, and LDLRAD4-AS1 genes with diagnostic significance were selected for prediction modelling and column Nomogram line plotting using Rtudio software. The diagnostic equation built for model was Logit(P)=7.43 + 1.71*(LDLRAD4_AS1)+0.57*(RP11_295G20.2)+0.79*(RP3_508I15.21) based on the basis of multivariable logistic regression and the result was displayed as nomogram shown in **Figure 8**. In the nomogram, each variable has a corresponding score according to the value, which was read out by drawing a line straight upward from each predictor to the point axis, and after calculating the total scores of the 3 variables, the risk of diagnosing a patient as having sepsis was intuitively demonstrated.

### Evaluation of forecasting models

**Figure 9:**
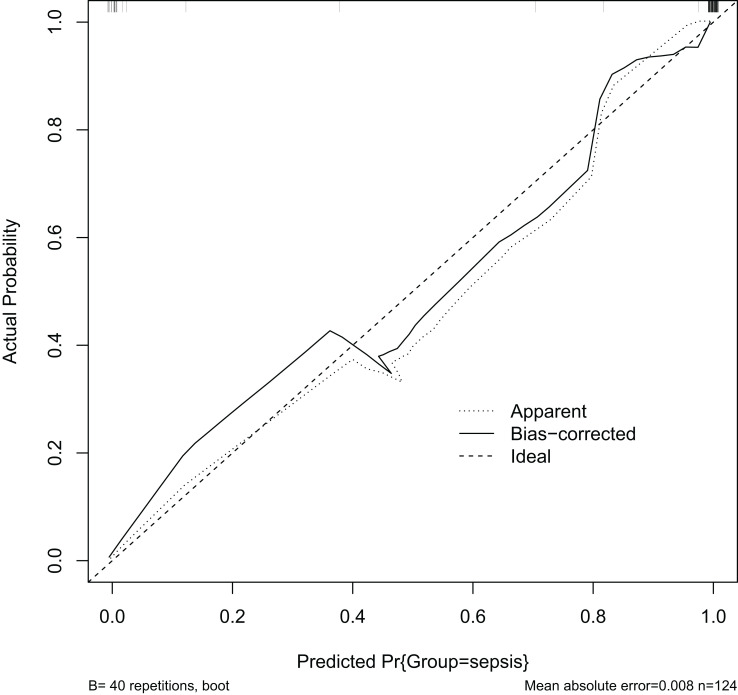
Internal calibration curve diagram.

Calibration of the prediction model is an important indicator for determining the predictive accuracy of the clinical prediction model. It indicates how well the model’s anticipated and actual values agree. A well-calibrated model has a high predictive accuracy, but a poorly calibrated model may exaggerate or underestimate the likelihood of an event. The calibration graph (**Figure 9**) shows a C-value of 0.73, indicating that the predictive model is effectively calibrated. The X-axis (Predicted Pr{Group=sepsis}) shows the likelihood of the model predicting sepsis. Y-axis (Actual Probability): indicates the actual probability of sepsis. Dashed line (Ideal): represents the calibration curve for the ideal case, i.e., the case where the predicted probability is exactly the same as the actual probability. The ideal line is a 45 degree diagonal. Solid (Bias-corrected): represents the calibration curve of the model after bias correction. This line shows how the model actually performs with different predicted probabilities. Dotted (Apparent): represents the model calibration curve without bias-correction.

### Evaluation of predictive models for clinical decision making

**Figure 10:**
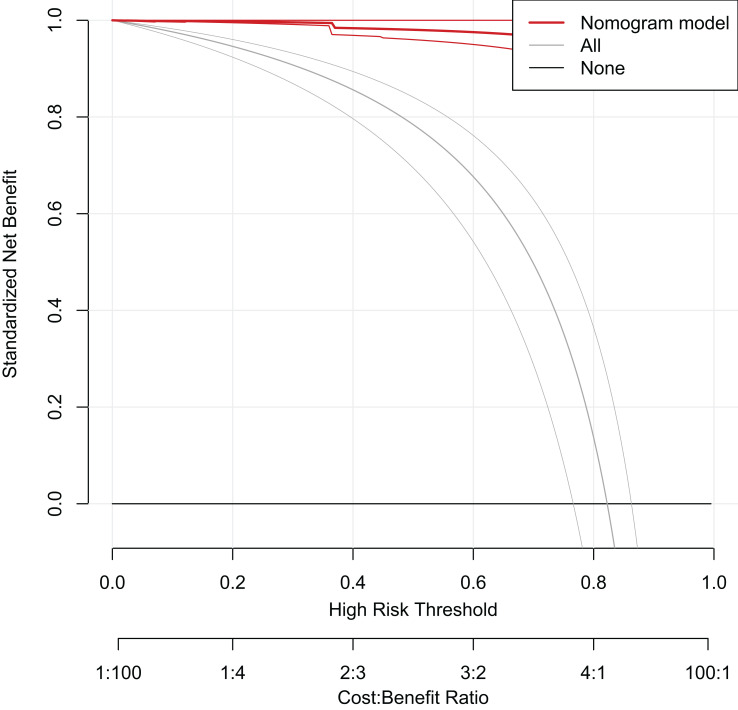
Decision curve analysis for predictive modeling.

To analyze the prediction model’s usability and performance, decision curve analysis (DCA) was used, and the decision curve was drawn using the rmda package in Rtudio program, as shown in **Figure 10**. The Y-axis (Standardized Net Benefit) displays the net benefit of the model at various risk thresholds. X-axis (High Risk Threshold): The High Risk Threshold shows the probability that the model will be classified as high risk. The X-axis (High Risk Threshold) displays the probability threshold for the model being forecasted to be high risk. Second X-axis (Cost: Benefit Ratio): The cost-benefit ratio, which compares the cost of a test or therapy to the benefit. Line Descriptions: The red line (Nomogram model) represents the net benefit of utilizing the Nomogram model. Grey line (All): shows the net benefit if all patients are treated. The black line (None) represents the net benefit assuming no patients were intervened in.

**Figure 11:**
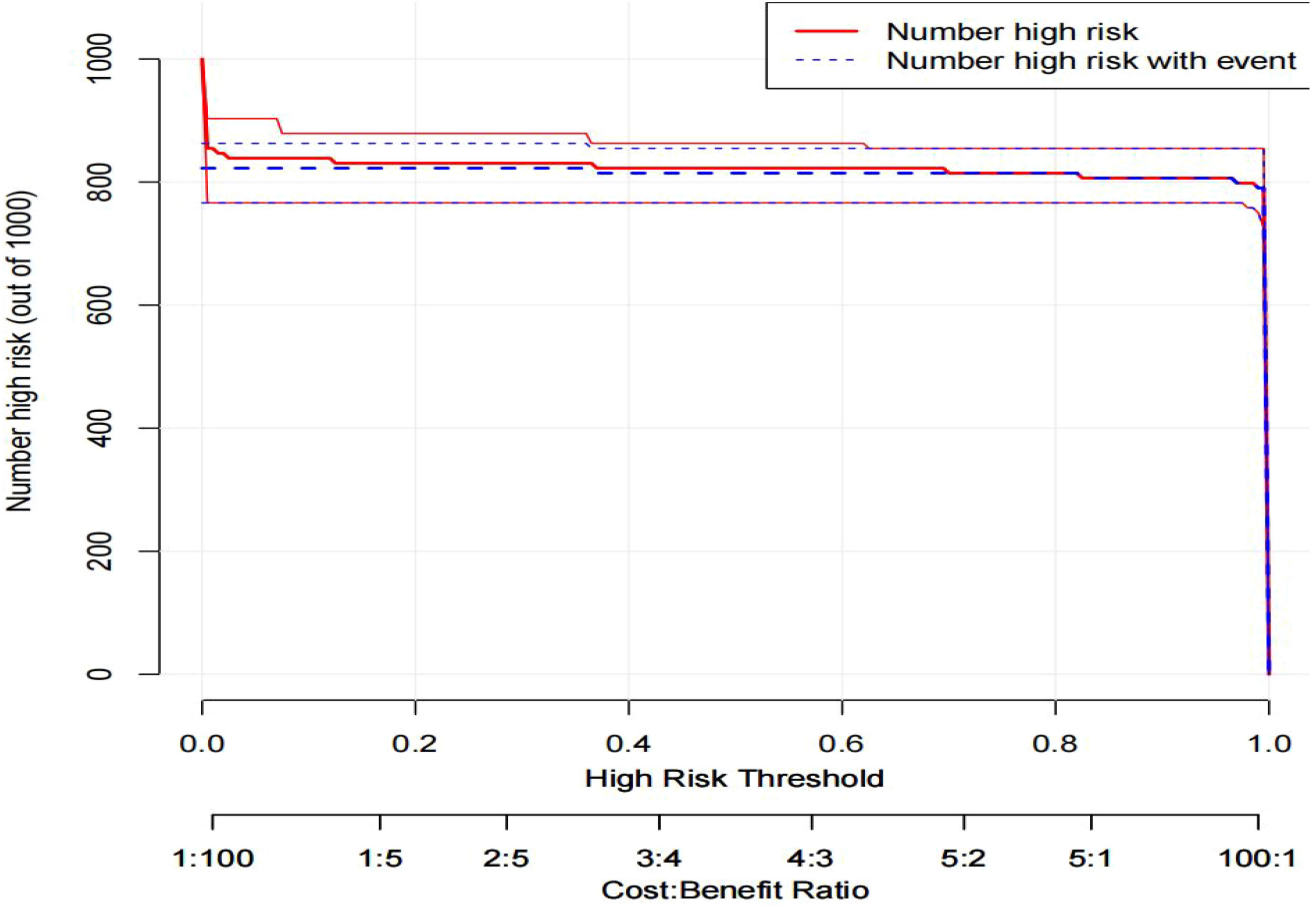
The clinical impact curve of the nomogram prediction model.

Clinical impact curves were constructed using DCA to analyze the number of patients classified as high risk based on the column line graph, as well as the number of true positive patients at each risk threshold. As shown in **Figure 11**, the X-axis (High Risk Threshold) displays the high risk threshold, which ranges from 0 to 1.This threshold establishes the parameters for a person to be classified high risk. The y-axis [Number high risk (out of 1000)] shows how many persons out of 1000 are considered high risk. The red solid line (Number high risk) represents the total number of people classified as high risk at various high risk levels. The blue dotted line (Number high risk with event) represents the total number of patients classified as high risk with actual sepsis at various high risk levels.

## Discussion

In recent years, researchers have focused on the relevance of lncRNAs in sepsis patients. lncRNAs can affect the inflammatory response by modulating the expression of inflammatory mediators (9–11). Certain lncRNAs can modulate inflammatory variables including IL-6 and TNF-α (12, 13), impacting the course of sepsis. LncRNAs play a crucial role in the formation and function of immune cells. They can alter the behavior of macrophages, T cells, and B cells, which in turn influences the body’s immunological response to infection (14, 15). Complex linkages between the inflammatory response and immunological control in sepsis. Sepsis causes immune cells to release cytokines such TNF-α, IL-1, and IL-6, resulting in systemic inflammation. Activation of the complement system increases the inflammatory response, causing tissue damage (16, 17). Cytokine storms create positive feedback loops that continuously activate the immune system and exacerbate the inflammatory response (18, 19). In the early stages of sepsis, the patient’s immune system is engaged and hyperactive; however, as sepsis progresses, high levels of inflammatory substances cause death of immune cells, particularly monocytes and T cells, and the body enters an immunosuppressive state (20–22). Immune cells regulate lncRNAs, which influence illness changes in sepsis patients. The ability of lncRNAs to modulate the processes of apoptosis and autophagy is critical for controlling cellular damage and tissue harm in sepsis (23, 24). Certain lncRNAs show drastically changed expression levels in the blood of sepsis patients and can be used as biomarkers for diagnosis and prognosis. For example, alterations in the levels of lncRNA HOTAIR and MALAT1 in sepsis patients were investigated (25).

In this work, we studied and analyzed using bioinformatic analysis approach and determined that lncRNA RP3_508I15.21, RP11_295G20.2, and LDLRAD4_AS1 were closely connected to sepsis.RP11_295G20.2 controls gene expression by interacting with chromatin remodeling complexes that control genes involved in cell proliferation, death, and stress responses (26). RP11_295G20.2 promotes tumor cell proliferation and survival by influencing the expression of these genes in research on cancer and other proliferative illnesses (27). Relevant investigations have demonstrated that RP11_295G20.2 can influence the levels of inflammatory mediators such as IL-6 and CRP via modulating the expression of inflammation-related genes (28). Regulates the expression of these mediators, which determines the inflammatory response and course of sepsis.LDLRAD4_AS1 influences the equilibrium of lipid metabolism *in vivo* by regulating the expression of lipid metabolism-related genes, such as the low-density lipoprotein receptor (LDLR) and its associated proteins (29). In metabolic disorders like as atherosclerosis and obesity, LDLRAD4_AS1 influences lipid metabolism pathways through the modulation of low-density lipoprotein receptor-associated protein 4 (LDLRAD4) expression (23). In immune-related diseases, such as sepsis and autoimmune disorders, LDLRAD4_AS1 may influence the strength and efficacy of immune responses by modulating immune cell behavior (24). There are few research on RP3_508I15.21, however the author’s current study discovered that RP3_508I15.21 was much higher in the sepsis group than in the non-sepsis group, and the difference was statistically significant. This research group believes that RP3_508I15.21, by directly or indirectly modulating key molecules in the inflammatory signaling pathway, may affect the intensity and duration of inflammation, as well as the differentiation, activation, and migration of immune cells and their function in the immune response, thereby regulating inflammation expression and influencing the immune response in sepsis. Further proof is required in animal or prospective research.

The methods of action of these lncRNAs in various diseases are complicated and diverse, primarily controlling gene expression, influencing inflammatory mediator levels, and modulating immune cell activity. Although existing research has offered some early evidence, the specifics of these systems require additional investigation. Future research is likely to disclose more about the specific mechanisms underlying these lncRNAs in sepsis. These lncRNAs’ unique expression alterations during sepsis, inflammatory response, and immunological damage have the potential to serve as diagnostic and prognostic markers. By focusing on these lncRNAs, new therapeutic techniques can be created to control inflammatory and immunological responses, enhancing the treatment of sepsis and other disorders. Studying the expression patterns of these lncRNAs in various patients would help to personalize treatment and increase its precision and effectiveness.

With the rapid development of multi-omics technology, such as genomics, proteomics, transcriptomics, immunomics, and others, clinical applications of lncRNA in sepsis patients are now available. LncRNA regulates gene expression, the inflammatory response, and immunological control, which can provide in-depth insights into pathophysiological mechanisms. LncRNA can also be detected in bodily fluids such as blood, making it suited for non-invasive diagnostics. LncRNAs can exhibit distinct expression patterns in sepsis and have good diagnostic and prognostic value. However, there are limitations. lncRNAs are diverse and functionally complicated, and their expression varies significantly across individuals and illness stages, complicating model building. Many lncRNAs’ biological functions remain unknown, limiting their usefulness as biomarkers.

## Conclusion

LncRNA RP3_508I15.21, RP11_295G20.2, and LDLRAD4_AS1 have some usefulness in the diagnosis and treatment of adult sepsis patients, and they may be useful in guiding the precise diagnosis and treatment of clinical sepsis.

## Data Availability

The datasets presented in this study can be found in online repositories. The names of the repository/repositories and accession number(s) can be found in the article/supplementary material.
